# Electrochromic Performance of V_2_O_5_ Thin Films Grown by Spray Pyrolysis

**DOI:** 10.3390/ma13173859

**Published:** 2020-09-01

**Authors:** Kyriakos Mouratis, Valentin Tudose, Cosmin Romanitan, Cristina Pachiu, Oana Tutunaru, Mirela Suchea, Stelios Couris, Dimitra Vernardou, Koudoumas Emmanouel

**Affiliations:** 1 Center of Materials Technology and Photonics, School of Engineering, Hellenic Mediterranean University, 71410 Heraklion, Greece; tudose_valentin@yahoo.com (V.T.); dvernardou@hmu.gr (D.V.); 2 Department of Physics, University of Patras, 26500 Patras, Greece; couris@physics.upatras.gr; 3 Department of Chemistry, University of Crete, 70013 Heraklion, Greece; 4 National Institute for Research and Development in Microtechnologies—IMT Bucharest, 126A, Erou Iancu 8 Nicolae Street, 077190 Bucharest, Romania; cosmin.romanitan@imt.ro (C.R.); cristina.pachiu@gmail.com (C.P.); oana.tutunaru@imt.ro (O.T.); 5 Department of Electrical and Computer Engineering, School of Engineering, Hellenic Mediterranean University, 71410 Heraklion, Greece

**Keywords:** vanadium pentoxide, electrochromic, spray pyrolysis, ammonium metavanadate

## Abstract

A new approach regarding the development of nanostructured V_2_O_5_ electrochromic thin films at low temperature (250 °C), using air-carrier spray deposition and ammonium metavanadate in water as precursor is presented. The obtained V_2_O_5_ films were characterized by X-ray diffraction, scanning electron microscopy and Raman spectroscopy, while their electrochromic response was studied using UV-vis absorption spectroscopy and cyclic voltammetry. The study showed that this simple, cost effective, suitable for large area deposition method can lead to V_2_O_5_ films with large active surface for electrochromic applications.

## 1. Introduction

Currently, many efforts are going on worldwide to develop new electrochromic materials for energy and environmental applications. V_2_O_5_ compounds keep attracting much attention because of their structural flexibility and chemical/physical properties, which are suitable for catalytic and electrochemical applications [1,2,3]. The layered structure with orthorhombic symmetry of α-V_2_O_5_ can be well adapted to the reversible incorporation of guest Li^+^ ions. As a result, its films have been recognized as the most attractive material for applications in electrochromic devices and in lithium batteries [3,4,5,6,7,8,9,10,11]. The valence state of V atoms is known to be extremely sensitive to the chemical environment, a behavior that can lead to a variety of structures containing different building units and exhibiting different physical properties. In particular, the structure and the morphology of vanadium oxide films are intimately related to the deposition method and the operating conditions. Moreover, since the direct growth of crystalline V_2_O_5_ films is very difficult except in the cases of some sub-stoichiometric oxides, annealing of the as-prepared films might be required in order to improve properties and functionality. Over the years, many techniques were involved for the deposition of V_2_O_5_ thin films for electrochromic applications, including electrodeposition, sputtering, sol-gel, hydrothermal growth and doctor-blade [12,13,14,15,16]. Some trials have been also reported on the use of spray pyrolysis for the deposition of large surfaces electrochromic V_2_O_5_ films, most of them using relatively high temperatures (>500 °C) vanadium chloride (i.e., moisture sensitive and toxic), vanadium nitride (i.e., irritant) as precursors [17,18,19,20].

The present letter concerns preliminary results on a new approach for developing nanostructured V_2_O_5_ thin films for electrochromic applications. In particular, air-carrier spray deposition at quite low temperature has been employed with ammonium metavanadate dissolved in water, which is a rather simple and low-cost precursor.

## 2. Materials and Methods

### 2.1. Materials

Ammonium metavanadate, ACS reagent, ≥99.0% (NH_4_VO_3_) was purchased from Sigma-Aldrich (St. Louis, MO, USA) and distilled water was used as a solvent for the precursor’s preparation. Lithium perchlorate, ACS reagent, ≥95.0% (LiCLO_4_) from Sigma-Aldrich was used as electrolyte and propylene carbonate, anhydrous, 99.7% from Sigma-Aldrich was the electrolyte’s solvent. Finally, fluorine-doped tin Oxide (FTO–SnO_2_:F) coated glass was the substrate.

### 2.2. Preparation of V_2_O_5_ Samples

A custom-made spray pyrolysis technique was utilized for the preparation of the samples. The deposition was carried out at a distance of 30 cm between the spray gun and the substrate, which was kept at a temperature of 250 °C. The precursor solution was prepared by dissolving the required amount of ammonium metavanadate (NH_4_VO_3_) in distilled water. Two concentrations were used, 0.01 M and 0.02 M. Since the optical difference between the colored and the bleached states of the as deposited samples was very low as well as their electrochromic performance was quite poor, thermal annealing was carried out for 2 h at 400 °C in oxygen atmosphere. More details regarding the sample names and the growth parameters can be found in Table 1. The respective thickness was measured to be about a micron.

### 2.3. Structural and Morphological Characterization

X-ray diffraction (XRD), scanning electron microscopy (SEM) and Raman Spectroscopy were used to analyze the structure and the morphology of the deposited V_2_O_5_ films. For the XRD measurements, a 9kW SmartLab X-ray Diffraction System (Rigaku, Tokyo, Japan) was employed, with rotating anode that employs Cu Kα1 radiation (λ = 1.5406 Å) in parallel-beam mode. To reveal the crystalline structure of the layer, the grazing incidence technique was used, the incidence angle was kept at 0.5°, while the detector was moved from 5 to 95°. SEM characterization was performed using a Nova NanoSEM 630 (FEI Company, Hillsborough, OR, USA), field emission scanning electron microscope for the annealed samples so that a better resolution and insight on their fine surface structuring can be obtained. All samples were characterized in high vacuum mode without any coating. Raman spectroscopy was performed at room temperature using a WiTec Raman spectrometer (Alpha-SNOM 300 S, WiTec GmbH, Ulm, Germany) using 532 nm excitation, from a diode-pumped solid-state laser with a maximum power of 145 mW. The incident laser beam with a spot-size of about 1.0 µm was focused onto the sample with a 100× long working distance microscope objective. The Raman spectra were collected with an exposure time of 20 s accumulation and the scattered light was collected by the same objective in back-scattering geometry using a 600 grooves/mm grating. The spectrometer scanning data collection and processing were carried out using the WiTec Project Five software (Version, WiTec GmbH, Ulm, Germany).

### 2.4. Electrochemical Characterization

Electrochemical experiments were performed using a PGSTAT302N Autolab (Metrohm AG, Herisau, Switzerland) potentiostat/galvanostat in a three-electrode cell setup, the working electrode with the V_2_O_5_ layer on the FTO substrate, the Pt counter electrode and the reference electrode (Ag/AgCl) utilizing 1M, LiClO_4_ in propylene carbonate as electrolyte [21].

### 2.5. Optical Characterization

The optical properties of the samples were evaluated by UV-Vis transmittance spectra recorded with a UV-2401PC spectrophotometer (Shimadzu Corporation, Kyoto, Japan,), at a wavelength range of 200 nm to 1000 nm.

## 3. Results and Discussion

### 3.1. Morphological Characterization

**Figure 1:**
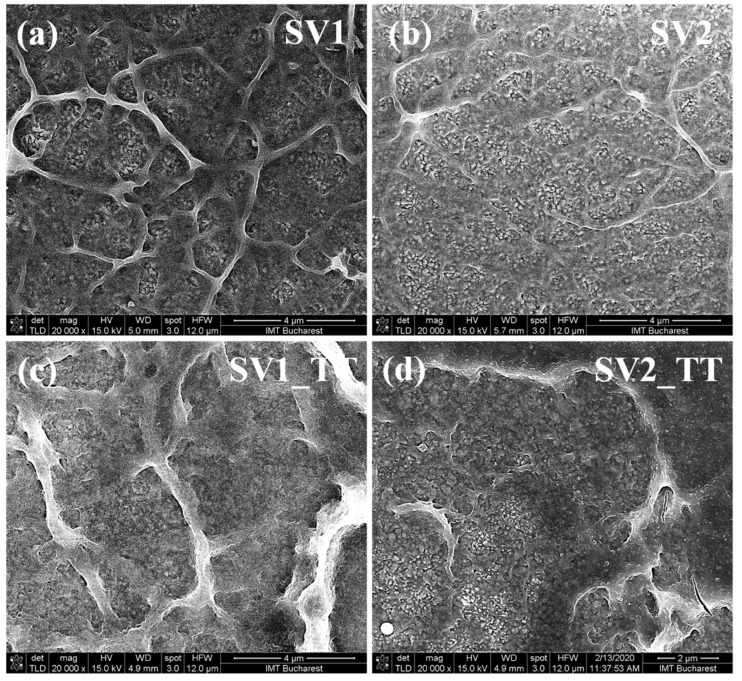
(**a**) SEM image of SV1 sample, (**b**) SEM image of SV2 sample, (**c**) SEM image of SV1_TT sample, (**d**) SEM image of SV2_TT sample.

As shown in Figure 1a,b, SEM characterization revealed the formation of mixed nanostructured granular and wall-like structures films, homogeneously distributed over the film surface.

Moreover, as can be observed, the films present a very large active surface when compared with usual flat granular structured films. Annealing of the films for 2 h at 400 °C in an oxygen atmosphere was found to lead to a decrease of the number of film surface features, as can be seen in Figure 1c,d. This kind of structuring has not been reported before when using other growth techniques and it seems to be directly related to the growth technique used in our work. The particular surface structuring and morphology makes these films suitable for applications that require a high surface to volume ratio.

### 3.2. X-ray Diffraction Analysis

**Figure 2:**
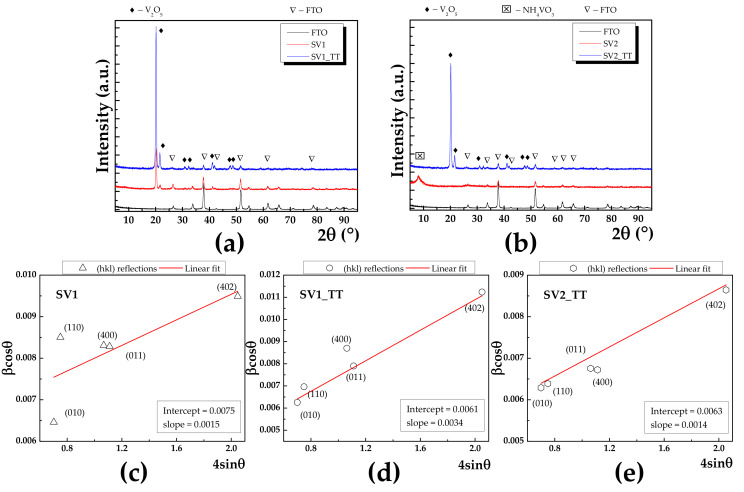
GIXRD patterns for (**a**) SV1 before and after annealing, (**b**) SV2 before and after annealing with red and blue line, respectively. In addition, GIXRD pattern corresponding to FTO was added (black line). (**c**–**e**) Williamson-Hall plots for SV1, SV1_TT and SV2, respectively with corresponding values for intercept and slope as inset.

Grazing incidence X-ray diffraction (GIXRD) was used to reveal the crystalline structure of the investigated samples. Since GIXRD requires small incident angles for the incoming X-rays, the diffraction is surface sensitive, the wave penetration being limited, and this approach is used for the study surfaces and layers. Figure 2a shows the GIXRD patterns of SV1 and SV2 before (red line) and after annealing (blue line).

In addition, the corresponding GIXRD pattern of the FTO substrate was also added (black line). The peak indexing was made using International Center for Diffraction Database (ICDD) database. In the case of SV1, the presence of the V_2_O_5_ phase was identified, with orthorhombic lattice having parameters a = 11.7 Å, b = 4.40 Å and c = 3.45 Å, while α = β = γ = 90°, which belongs to the 47:Pmmm space group. To gain a thoroughly view of the crystalline features of our materials, we employed Williamson-Hall method, which allowed us to separate the strain and size effects from the Bragg peak. The formalism behind this method can be found in [22]. Figure 2c–e present the Williamson-Hall plots (red line) for SV1, SV1_TT and SV2_TT with corresponding intercept and slope values as inset, which are related to the mean crystallite size and lattice strain, respectively. As a result, the mean crystallite size was increasing from 18.4 to 22.7 nm. For the respective as deposited SV2 sample, the XRD pattern exhibited only a broad peak at 8.07°, which can be attributed to the non-reacted precursor NH_4_VO_3_ since it cannot be matched with any vanadium oxide phase. After annealing, the occurrence of V_2_O_5_ was visible and the respective mean crystallite size calculated to be 22.1 nm. At the same time, after the annealing process, the lattice strain ε increases from 0.15% (e.g., SV1) to 0.34% (e.g., SV1_TT), which indicates a slight expansion of the interplanar distance. The XRD results indicate that annealing can result in two main effects.

Firstly, the crystallinity is enhanced, which leads to a smaller dislocation density, whose occurrence is due to the boundaries of the adjacent mosaic blocks [23]. Simultaneously, the decreasing of dislocation density is accompanied by a slight expansion of the lattice strain. Secondly, the full conversion from NH_4_VO_3_ to V_2_O_5_ phase can take place.

### 3.3. Raman Analysis

**Figure 3:**
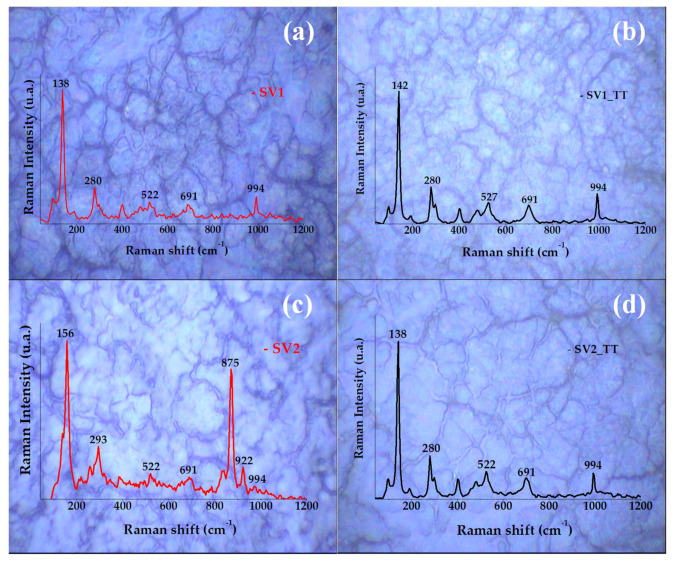
Raman spectra overlapped over optical microscopy images (100× magnification) of SV1 (20 mL, 0.02 M) and SV2 (20 mL, 0.01 M) samples, (**a**,**c**) before annealing, (**b**,**d**) after annealing.

Since the structure of α-V_2_O_5_ (orthorhombic) belongs to the Pmmn space group [24,25], the V_2_O_5_ layers are formed from packing of edge shared VO_5_ square pyramids linked in the ‘ΧΥ’ plane. Group theoretical analysis predicts twenty-one Raman active modes for V_2_O_5_ at Γ point, 7Ag + 7B2g + 3B1g + 4B3g [26,27]. In our case we observed five Raman modes for the annealed films as shown in Figure 3, which match with the reported data for V_2_O_5_.

In particular, the Raman peaks we observed were at 138, 280, 522, 691 and 994 cm^−1^, confirming the presence of α-V_2_O_5_ phase [26], as was already identified with XRD. The 5 observed Raman peaks can be assigned as follows:

(a) The highest frequency peak at 994 cm^−1^ appears to be due to the stretching vibrational mode of V–OI bond along Z direction.
(b) Displacement of OIII atoms in Y and X directions generates Raman modes at 691 cm^−1^ (V–OIII–V antiphase stretching mode) and 522 cm^−1^ (d4 stretching vibration), respectively.
(c) Mode 280 cm^−1^ can be attributed to oscillation of OI atoms along Y axis.
(d) The low frequency mode at 138 cm^−1^ corresponds to Y displacement of the whole chain involving shear and rotations of the ladder like V–OIII bonds. The high intensity of 138 cm^−1^ peak indicates the long range order of V–O layers in the XY plane.

Considering the annealing effect on the film structuring, the most affected modes by the temperature were found to be the V^+2^=O stretching bands situated at 138 cm^−1^ which has a slight deviation to the left and the V^+3^=O situated at 522 cm^−1^ which increases in Raman intensity. The other V_2_O_5_ vibration modes (V^+3^=O stretching modes situated at 691 cm^−1^ and V^+5^=O stretching modes situated at 994 cm^−1^) were not affected by the compressive stress introduced by temperature [28]. Supplementary peaks associated to nonreacted metavanadate precursor, were detected at 871 cm^−1^ in the SV2 current study.

### 3.4. Electrochemical Characterization

**Figure 4:**
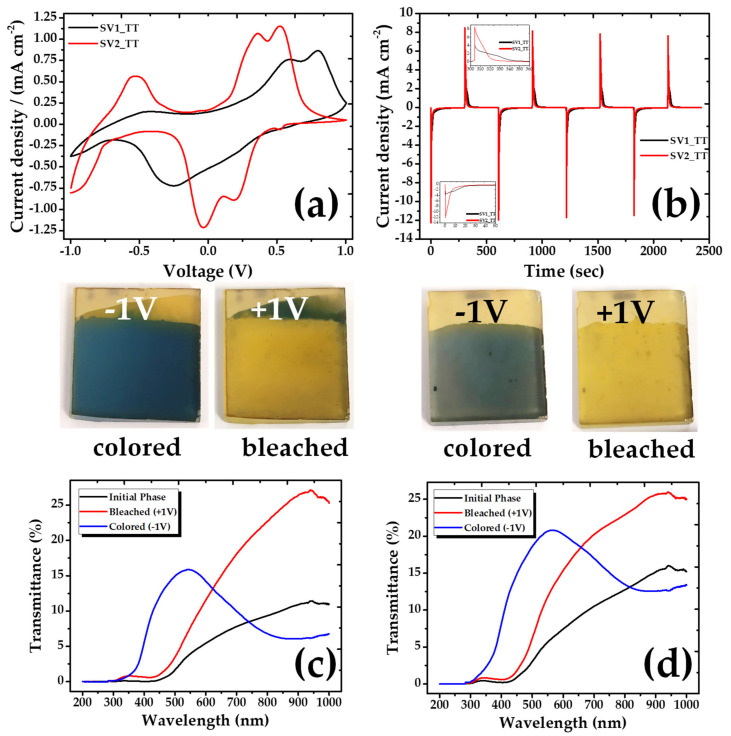
(**a**) Cyclic voltammograms after annealing, (**b**) Chronoamperometry after annealing, (**c**,**d**) UV-Vis transmittance comparison of the initial, bleached and colored state of the V_2_O_5_ coatings, (**c**) SV1_TT, (**d**) SV2_TT.

The electrochemical performance of the samples was studied in a three-electrode cell, by cycling the potential between −1 V and +1 V at a scan rate of 10 mV s^−1^ and using 1M, LiClO_4_ in propylene carbonate as electrolyte (Figure 4a).

In parallel, their electrochromic performance was studied with UV-Vis absorption spectroscopy. A minimal optical difference between colored and bleached states of the as deposited samples along with poor electrochromic properties were indicated. In particular, the color varied between light gray/yellow (−1 V) and light yellow (+1 V). In order to improve the structure of the V_2_O_5_ films and their subsequent performance, the samples were annealed by heating them in an oven at a temperature of 400 °C for 2 h in an oxygen atmosphere. After annealing, the color states changed and improved dramatically, being blue for the SV1 sample and a darker gray for the respective SV2, as compared to the original colors before the annealing process. Comparing the bleached states, the annealed samples have stronger yellow tint than the samples before the annealing. The color changes of the annealed samples are presented in Figure 4.

The SV2_TT coating indicates three cathodic peaks at −0.926 V/−0.010 V/+0.230 V and three anodic peaks at −0.634 V/+0.328 V/+0.485 V, which are attributed to Li^+^ intercalation and deintercalation accompanying gain and loss of an e^−^. The current density of the particular sample is the highest indicating an enhanced electrochemical activity. On the other hand, the curve of SV1_TT is different showing two anodic and one cathodic peak (Figure 4a). This discrepancy may be related to the expansion of the interplanar distance of SV2_TT, which allows accommodating a greater number of Li ions.

Chronoamperometry measurements were used to evaluate the time evolution of the coloring/bleaching processes (Figure 4b). When a negative voltage (−1.0 V) was applied, the color of the V_2_O_5_ coating changed from yellow to blue (SV1_TT sample) or dark grey (SV2_TT sample). With the opposite applied voltage (+1.0 V), the film returned to its initial state, becoming yellow again. From the I–t measurements, the time responses for coloration of the SV1_TT and SV2_TT samples were found to be 34.8 s and 9.8 s, respectively, the respective times for bleaching being 34.2 s and 15.7 s. As can be observed, SV2_TT has a much faster response. The charge densities of these samples, calculated by integrating the current density, were found to be −73.3/−71.01 and 70.7/70.9 mC cm^−2^ for the SV1_TT and SV2_TT samples, respectively.

The transmittance measurements performed in the annealed samples are also shown in Figure 4c, d. In these graphs, we can observe how the transmittance spectra change between the colored and the bleached state according to the applied voltages in the samples. More specifically, it is obvious that above 650 nm, the color difference is increasing, a behavior indicating that the electrochromic performance of our samples is maximized at the near infrared spectral region. 

Finally, the coloration efficiency (*CE*) factor, defined as the change in optical density (Δ*OD*) per unit of charge density (*Q*), was calculated according to the following Equation (1):

(1) $$CE(\lambda )=\frac{\Delta OD(\lambda )}{Q}$$

where Δ*OD* is the optical density, showing the change in the transmission between the bleached and colored states of the film, that is, Equation (2):

(2) $$\Delta OD(\lambda )=\ln\left(\frac{T_{b}}{T_{c}}\right)$$

where, *T_b_* and *T_c_* are the transmittances at bleached and colored state, respectively. *Q* is the charge density and *λ* denotes certain wavelengths, which, for our calculations, were taken at 700 and 900 nm. The *CE* for our films was calculated to be 8.1 and 2.4 cm^−2^ C^−1^ for SV1_TT and SV2_TT respectively at 700 nm, increasing up to 20 and 10 cm^−2^ C^−1^ at 900 nm. Although the coloration efficiency is higher for the SV1_TT sample, the experiments showed that this is decaying faster than the SV2_TT one, which is presenting better crystalline properties. Since these are preliminary result, further efforts are currently under way in order to increase both the coloration efficiency and the stability of the films.

## 4. Conclusions

Electrochromic nanostructured V_2_O_5_ thin films were prepared at low temperature (250 °C) using air-carrier spray deposition, starting from ammonium metavanadate precursor in water. The obtained V_2_O_5_ films were characterized by X-ray diffraction, scanning electron microscopy and Raman spectroscopy, while their electrochromic behavior was studied using UV-vis absorption spectroscopy and cyclic voltammetry. This preliminary study showed that this simple, cost effective, suitable for large area deposition method can lead to novel surface structuring of V_2_O_5_ films with electrochromic performance. Further studies for growth optimization and improvements of film properties and stability would be performed.

## Figures and Tables

**Table 1:** Growth parameters of V_2_O_5_ thin films.

Sample Name	Substrate	Precursor Volume (mL)	Temperature (°C)	Concentration (M)	Annealing
SV1	FTO	20	250	0.02	No
SV2	FTO	20	250	0.01	No
SV1_TT	FTO	20	250	0.02	400 °C for 2 h
SV2_TT	FTO	20	250	0.01	400 °C for 2 h
